# Test–Retest Reliability of Magnetoencephalography Resting-State Functional Connectivity in Schizophrenia

**DOI:** 10.3389/fpsyt.2020.551952

**Published:** 2020-12-16

**Authors:** Felicha T. Candelaria-Cook, Julia M. Stephen

**Affiliations:** The Mind Research Network, Albuquerque, NM, United States

**Keywords:** schizophrenia, MEG, test–retest reliability, resting-state, functional connectivity

## Abstract

The reliability of magnetoencephalography (MEG) resting-state functional connectivity in schizophrenia (SZ) is unknown as previous research has focused on healthy controls (HC). Here, we examined reliability in 26 participants (13-SZ, 13-HC). Eyes opened and eyes closed resting-state data were collected on 4 separate occasions during 2 visits, 1 week apart. For source modeling, we used minimum norm software to apply dynamic statistical parametric mapping. Source analyses compared the following functional connectivity metrics from each data run: coherence (coh), imaginary coherence (imcoh), pairwise phase consistency (ppc), phase-locking value (plv), phase lag index (pli), weighted phase lag index (wpli), and weighted phase lag index debiased (wpli2). Intraclass correlation coefficients (ICCs) were calculated for whole brain, network, and network pair averages. For reliability, ICCs above 0.75 = excellent, above 0.60 = good, above 0.40 = fair, and below 0.40 = poor reliability. We found the reliability of these metrics varied greatly depending on frequency band, network, network pair, and participant group examined. Broadband (1–58 Hz) whole brain averages in both HC and SZ showed excellent reliability for wpli2, and good to fair reliability for ppc, plv, and coh. Broadband network averages showed excellent to good reliability across 1 hour and 1 week for coh, imcoh, ppc, plv, wpli within default mode, cognitive control, and visual networks in HC, while the same metrics had excellent to fair reliability in SZ. Regional network pair averages showed good to fair reliability for coh, ppc, plv within default mode, cognitive control and visual network pairs in HC and SZ. In general, HC had higher reliability compared to SZ, and the default mode, cognitive control, and visual networks had higher reliability compared to somatosensory and auditory networks. Similar reliability levels occurred for both eyes opened and eyes closed resting-states for most metrics. The functional connectivity metrics of coh, ppc, and plv performed best across 1 hour and 1 week in HC and SZ. We also found that SZ had reduced coh, plv, and ppc in the dmn average and pair values indicating dysconnectivity in SZ. These findings encourage collecting both eyes opened and eyes closed resting-state MEG, while demonstrating that clinical populations may differ in reliability.

## Introduction

Magnetoencephalography (MEG) is an advantageous neuroimaging tool to study psychosis due to its safety as a non-invasive test, along with the high dimensional data it provides on neuronal activity, oscillatory dynamics, and connectivity at a millisecond time scale. The spontaneous oscillatory signals captured by MEG during a resting-state can be used to estimate neural interactions between brain regions and reveal network disorganization and abnormalities in schizophrenia (SZ) and other clinical populations. Despite the increasing prevalence of resting-state MEG research, studies examining the reproducibility and reliability of MEG-derived functional connectivity measures remain scarce, especially in clinical populations where reliability is critical for clinical application.

Previous resting-state MEG test–retest reliability has been evaluated in healthy controls (HC) (1–5), patients with depression (6), and patients with SZ (5). Most test–retest studies use intraclass correlation coefficients (ICCs) or Spearman correlations to report and categorize degree of reliability. To model and interpret an ICC, with values ranging from 0 to 1, excellent reliability is defined as ICC > 0.75, good reliability ICC = 0.75-0.60, fair reliability ICC = 0.59–0.40, and poor reliability ICC < 0.40 (7). In HC, MEG spectral power has good reliability in theta, alpha, and beta bands (ICCs > 0.6) over a 7 day test–retest interval (3) and excellent reliability in theta-gamma bands (ICCs > 0.86) in global and regional spectral measures over both 1 hour and 1 week test–retest intervals (5). The reliability of MEG functional connectivity has varied greatly in HC depending on the connectivity metric used (1, 2, 5) and frequency band studied (4). For example, the reliability of phase-locking value (plv) in alpha, beta, and gamma bands average between ICCs = 0.74–0.82, but dip to ICCs < 0.1 when phase-lag index (pli) is used (2). Conversely, other studies have reported weighted phase-lag index (wpli) and the imaginary part of coherency had excellent to good reliability of global connectivity over 30 trials in alpha and theta bands, but often fair to poor reliability in other frequency bands and in vertex-based connectivity (4). It is clear given the variability of previous MEG functional connectivity findings that more research is needed to reach a consensus on which functional connectivity metric is best suited for MEG resting-state studies.

In clinical populations much less is known about the reliability of MEG functional connectivity metrics. Examining resting-state functional connectivity in patients with SZ can be especially informative given that SZ is often conceptualized as a disorder of altered brain connectivity by the disconnection hypothesis (8) with abnormal resting-state brain networks demonstrating disorganization (9). MEG functional connectivity abnormalities in patients with SZ, quantified by imaginary coherence (imcoh), include decreased left prefrontal cortex and right superior temporal cortex connectivity in alpha band which negatively correlated with negative symptoms, together with increased connectivity in left extrastriate cortex and right inferior prefrontal cortex (10). Other studies using spatial independent component analysis and pairwise correlations found hyperconnectivity within frontal and temporal networks in patients with SZ (11), information which was valuable in improving classification when combined with fMRI functional connectivity (12), in addition to hypoconnectivity between sensorimotor and task positive networks in the delta frequency band (13). Patients with SZ also have shown abnormalities in dynamic functional connectivity by changing meta-states more often than HC and exhibiting greater inter-individual variability (14), metrics which correlated with positive symptoms (15). For a more complete review of MEG abnormalities reported in SZ please refer to the following review papers (16–18). These previous findings, however, have not been replicated or shown test–retest reliability.

Recently we examined the 1 week reliability of MEG resting-state spectral power in a cohort of patients with SZ and HC. Overall we found that spectral power measures (power, normalized power, alpha reactivity) had excellent reliability for both HC and SZ in 1) global power averages in theta-gamma bands, 2) for all frequency bands across sensor regions, and 3) within parietal regions for alpha frequency (5). Furthermore, for patients, higher PANSS positive scores were negatively correlated with reduced parietal alpha normalized power. We also briefly examined a single functional connectivity metric, weighted phase lag index debiased (wpli2), and found poor reliability for the metric in both groups (5). The current study was designed as a follow-up to further explore other functional connectivity metrics which may perform better in patients with SZ. Where the previous study provided an in-depth analysis of MEG spectral power in patients with SZ and HC, the current study aims to provide an in-depth analysis of MEG functional connectivity in patients with SZ and HC.

The current study was designed to determine the test–retest reliability of MEG resting-state functional connectivity over 1 hour and 1 week intervals in psychosis. As such, it is one of the first studies to directly address MEG functional connectivity reliability in patients with SZ. MEG resting-state data were collected in 13 patients with SZ and 13 matched HC. Data were collected across 1 week (2 visits, 2 runs per visit). Each MEG session analyzed included both a 10 min and a 4 min rest session with rest phase alternating between an eyes open and eyes closed state. We hypothesized reliability would be lower in the patient group, when compared to HC, and that certain connectivity metrics, such as wpli2 would have poor reliability, similar to our previous study (5). Furthermore, when directly comparing functional connectivity metrics, we expected patients with SZ to have reduced connectivity when compared to HC, in line with (16). This study compared the reliability of various functional connectivity metrics in source space across 1 hour and 1 week intervals, using coherence (coh), imcoh (imaginary coherence), pairwise phase consistency (ppc), plv (phase-locking value), pli (phase-lag index), wpli (weighted phase lag index), and wpli2 (weighted phase lag index debiased). To determine reliability, ICCs were calculated and compared for whole brain averages, network connectivity averages, and regional connectivity pairs. Furthermore, ICCs were compared between patients with SZ and HC to determine which measures were most stable in a patient population.

## Materials and Methods

### Participants

The current study used existing data from 13 individuals diagnosed with SZ and 13 HC, age and gender matched (5). All participants were within 21–49 years of age, Table 1, and were compensated for their participation. Participant characteristics and procedures will be briefly described here, for further information on methods please refer to (5). Based on the Structured Clinical Interview for DSM-IV-Patient (SCID-IP), SZ participants had a DSM-IV-TR diagnosis of SZ, along with retrospective clinical stability. Based on the Structured Clinical Interview for DSM-IV-Non-patient (SCID-NP), HC participants had no psychiatric or neurological disorders and no history of developmental delays. All participants gave their informed consent according to institutional guidelines. The University of New Mexico Health Sciences Center Human Research Review Committee approved this study. Although participant data and rest preprocessing procedures used here overlap with that presented in (5), the analytic approach used here, specifically focusing on comparing different functional connectivity metrics, is novel and distinct.

**Table 1:** Participant characteristics.

	**Healthy Controls (Mean ±SD)**	**Patients with Schizophrenia (Mean ±SD)**
**Demographics**		
Gender (M/F)	8/5	8/5
Age (Males)	32.65 ± 8.88	32.98 ± 7.18
Age (Females)	37.95 ± 6.51	38.35 ± 7.99
Education (years) ^**^	15.23 ± 2.13	13.00 ± 1.41
Ethnicity (% Hispanic)	23%	46%
Ethnicity (% non-Hispanic)	77%	54%
Duration of Illness (yrs)	—	15.00 ± 9.34
**Data quality**		
% Epochs rejected eyes open	3.07 ± 3.47	5.62 ± 3.31
% Epochs rejected eyes closed	1.67 ± 2.40	3.65 ± 6.82
% Epochs rejected total	2.39 ± 2.71	4.64 ± 2.56
Avg # epochs Rest10 total	286.85 ± 20.49	282.81 ± 28.85
Avg # epochs Rest4 total	114.92 ± 7.84	115.23 ± 10.62
Avg Euclidean distance Rest10	4.62 mm	6.06 mm
Avg Euclidean distance Rest4	4.90 mm	6.12 mm

Asterisks represent significant differences between groups (

** *p < 0.05)*.

*All other comparisons (p > 0.05)*.

### Structural MRI Data Acquisition

To map source locations structural MRIs were obtained after MEG scans. Sagittal T1-weighted anatomical MR images were acquired using a Siemens TIM Trio 3 Tesla MRI system with a 32-channel head coil. Parameters of the T1-weighted MPRAGE sequence were: TR = 2,530 ms, TE = 1.64 ms, 3.5 ms, 5.36 ms, 7.22 ms, 9.08 ms, TI = 1,200 ms, 1.0 mm slice thickness, 192 slices, 7° flip angle, field of view (FOV) = 256 mm × 256 mm, matrix = 256 × 256, GRAPPA acceleration = 2 (5).

### MEG Behavioral Tasks

**Figure 1:**
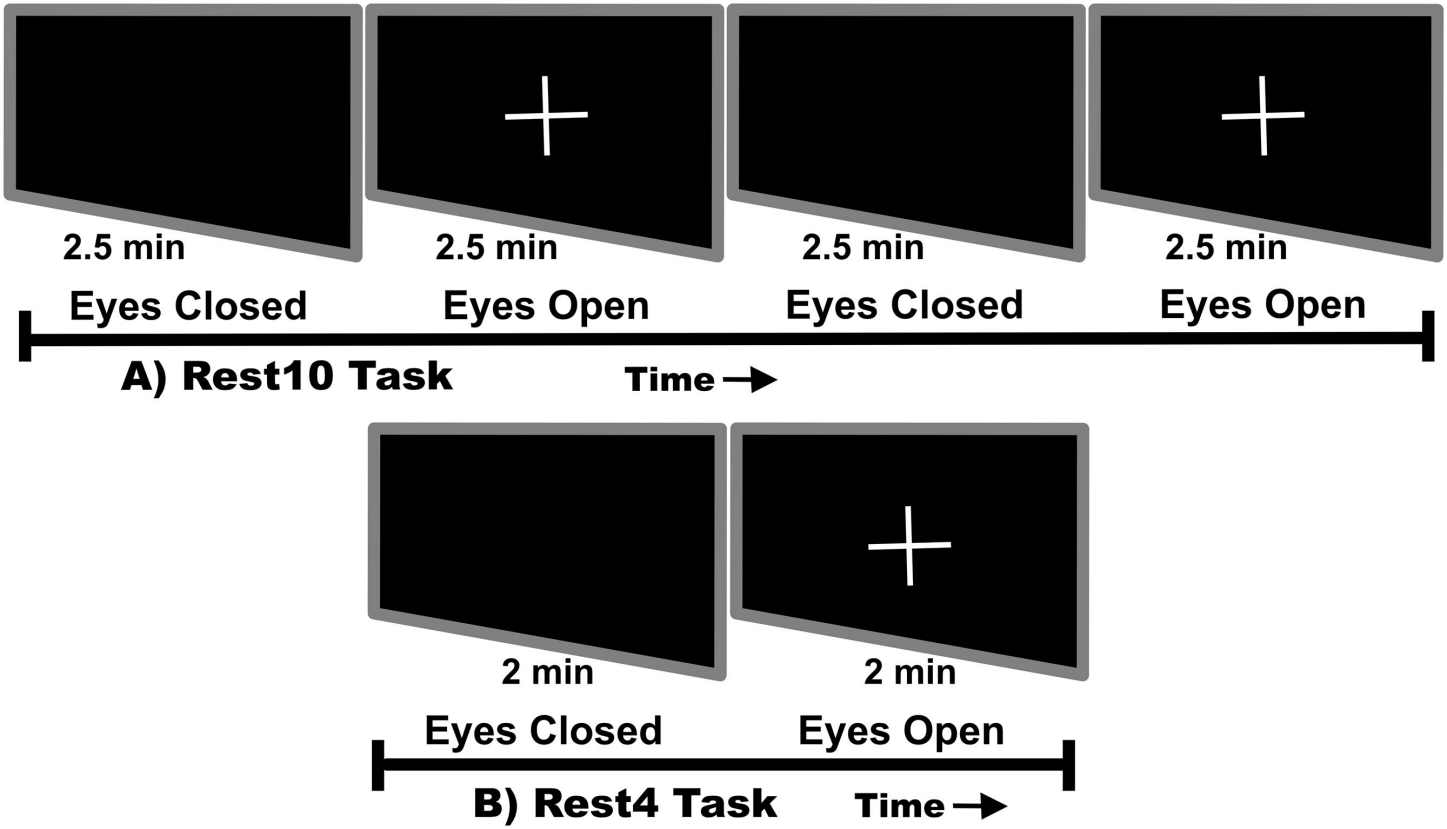
Rest task design. Rest10 alternates between 2.5 min of eyes closed followed by 2.5 min of eyes open fixated on a cross. Rest4 alternates between 2 min of eyes closed followed by 2 min of eyes open fixated on a cross.

Visits occurred 7 days apart. In order to avoid circadian rhythm influence on reliability, time of day was matched between visits. The average time for return visits for HC was 7.54 days ± 60 min and for SZ was 7.84 days ± 51 min. During each visit, the hour long MEG scan began with a 10-min rest task and ended with a 4-min rest task. At the start of each task participants were instructed to monitor prompts to close their eyes or open their eyes and fixate on a white cross. As shown in Figure 1, each task alternated between equal phases of eyes closed and eyes opened. The 10-min task, herein referred to as Rest10, changed phase every 2.5 min, while the 4-min task, herein referred to as Rest4, changed phase every 2 min. In total, resting-state activity was recorded during 4 separate runs (Visit1_Rest10, Visit1_Rest4, Visit2_Rest10, Visit2_Rest4).

### MEG Data Acquisition and Preprocessing

MEG data were collected with a 306-channel whole-head MEG system (Elekta Neuromag) in a magnetically shielded room (Vacuumschmelze—Ak3B) at the Mind Research Network in Albuquerque, New Mexico. Electro-oculogram and electrocardiogram channels were placed on the participant to monitor heartbeat and eyeblink artifacts. In addition, using three-dimensional digitization equipment (Polhemus FastTrack), four electromagnetic coils were registered to the nasion and preauricular points. During the tasks, data were sampled at 1,000 Hz and a Continuous Head Position Indicator (cHPI) was used to correct for motion. During each visit participants sat upright and head position was monitored closely. Average Euclidean distance was calculated for each task, see Table 1. Head position consistency was similar between HC and SZ (all *p*'s > 0.31) (5).

Using Neuromag MaxFilter 2.2 software, raw data were corrected for noise and head motion artifacts with the temporal extension of signal space separation (t-SSS) method with movement compensation (19, 20). For equivalent sensor locations, head position was transformed between visits with the MaxFilter 2.2, MaxMove option. Using signal space projection (SSP) (21) in MNE software (22), data were cleaned from heartbeat and eye-blink artifacts. Any data that failed the automated process was visually inspected and SSPs to remove artifacts were generated manually. After ensuring the data were artifact-free, continuous files were segmented into 2 sec epochs. Epochs were rejected if the magnetic field exceeded 5 pT. Data quality was equivalent between groups (all *p*'s > 0.25), see Table 1.

### MEG Source Analysis

Similar to previous processing (5), the cortical surface of each participant was reconstructed from T1-weighted MRI files using FreeSurfer. To create a source space of 4.9 m with 4,098 locations per hemisphere, a repeatedly subdivided octahedron was used as the spatial subsampling method. In MNE software (22, 23), dynamic statistical parametric mapping (dSPM) (24) was used to create an anatomically constrained linear estimation inverse model. The dSPM inverse model identified where the estimated current at each cortical surface vertex differed significantly from empty room data. Other data parameters were: depth weight of 0.8, loose constraint of 0.2, orientation of none, and signal-to-noise ratio of 3. A single layer (inner skull) boundary element method (25) was used to create the forward solution. A surface-based source space was used to confine source locations to a fixed surface orientation. When using a fixed source space, loose/free orientations are not normed, leading to signed source activity.

Source estimates were derived from epoch files. Using the FreeSurfer DKT parcellation (26, 27), average time series were extracted for 62 regional labels. The spectral connectivity computation performed in MNE software (version 0.19.0) (22, 23) used multitaper spectrum estimation with 7 DPSS windows. The frequency bands used were defined as: broadband (1–58 Hz), delta (1–4 Hz), theta (5–8 Hz), alpha (9–13 Hz), beta (14–29 Hz), and gamma (31–58 Hz). The connectivity methods extracted were: coh, imcoh, ppc, plv, pli, wpli, and wpli2. Since an aim of the study was to compare available functional connectivity metrics, not create, or modify existing ones, we report connectivity values produced by MNE without modification. In the case of imcoh, MNE uses the original definition (28), not the absolute value, to calculate imcoh values. The results of the spectral connectivity computation were run through custom scripts in MATLAB (2019a, MathWorks) to create whole brain, network, and regional pair averages. Whole brain values were derived from averaging all 62 regional labels, network values were derived from averaging regional labels within predefined clusters (29), and regional pair values were predefined 1-to-1 select regional connections. The labeling used between functional and anatomical regions is shown in Table 2. These resting-state networks are semi-independent anatomical clusters of correlated brain activity commonly examined during rest (29). Regional pairs were chosen from the default mode, cognitive control, and visual networks. The pairs represent either unilateral or contralateral connecting nodes within the same resting-state network. The default mode pair was contralateral right hemisphere precuneus to left hemisphere medial orbitofrontal region, the cognitive control pair was unilateral left hemisphere inferior parietal to left hemisphere caudal middle frontal region and the visual network pair was contralateral left hemisphere lateral occipital to right hemisphere middle temporal, Table 2. These regions were chosen to maximize regional distance within networks in an effort to minimize the effects of signal leakage on connectivity measures.

**Table 2:** Labeling between functional and anatomical regions.

**Network**	**Anatomical areas**	
Auditory	superior temporal-L	superior temporal-R
Somatosensory	paracentral-L	paracentral-R
	superior parietal-L	superior parietal-R
	postcentral-L	postcentral-R
	supramarginal-L	supramarginal-R
Visual	cuneus-L	cuneus-R
	lingual-L	lingual-R
	fusiform-L	fusiform-R
	middle temporal-L	middle temporal-R^*^
	lateral occipital-L^*^	lateral occipital-R
	pericalcarine-L	pericalcarine-R
Cognitive control	caudal middle frontal-L^*^	caudal middle frontal-R
	entorhinal-L	entorhinal-R
	inferior parietal-L^*^	inferior parietal-R
	inferior temporal-L	inferior temporal-R
	insula-L	insula-R
	lateral orbitofrontal-L	lateral orbitofrontal-R
	parahippocampal-L	parahippocampal-R
	pars opercularis-L	pars opercularis-R
	pars orbitalis-L	pars orbitalis-R
	pars triangularis-L	pars triangularis-R
	precentral-L	precentral-R
	rostral middle frontal-L	rostral middle frontal-R
	transverse temporal-L	transverse temporal-R
Default mode	caudal anterior cingulate- L	caudal anterior cingulate- R
	isthmus cingulate-L	isthmus cingulate-R
	medial orbitofrontal-L^*^	medial orbitofrontal-R
	posterior cingulate-L	posterior cingulate-R
	precuneus-L	precuneus-R^*^
	rostral anterior cingulate-L	rostral anterior cingulate-R
	superior frontal-L	superior frontal-R

*L, left hemisphere; R, right hemisphere*.

* *Denotes network pair*.

### Spectral Connectivity Estimation

Using MNE spectral connectivity commands, spectral connectivity was determined for the following 7 metrics: coh, imcoh, ppc, plv, pli, wpli, and wpli2. Coh is a generalization of correlation to the frequency domain, while imcoh is similar but is sensitive to synchronizations of two processes which are time-lagged to each other and avoids volume conduction artifacts by acknowledging that volume conduction does not cause a time-lag (28). Plv characterizes a stable phase relationship between two timecourses in a particular frequency band within a predefined window (i.e., rhythmic neuronal synchronization) (30). Ppc is very similar to plv, but is bias-free and consistent with population parameter statistics by using an equivalent to squared plv (31). Pli uses similar information but improves upon plv by disregarding zero-lag phase differences (32). Furthermore, pli quantifies the asymmetry of the phase difference distribution and estimates the likelihood for a consistent phase lead or lag between signals from two sensors. Wpli builds upon phase lag index by weighting observed phase leads and lags by the magnitude of the imaginary component of the cross-spectrum (33). These additions reduce sensitivity to uncorrelated noise sources while increasing power. Wpli2 is a debiased estimator of the squared wpli which corrects for sample-size bias in phase-synchronization indices (33). Of the 7 metrics used, 2 are considered spectral coherence metrics (coh, imcoh) and 5 are considered phase estimation metrics (plv, ppc, pli, wpli, wpli2). Furthermore, 4 are robust against spatial leakage artifacts (imcoh, pli, wpli, wpli2) and 3 are not leakage corrected (coh, plv, ppc). Because the goal of the study was to compare available functional connectivity metrics from an already available software package, values were reported without modification. The formulas used by MNE were according to original definition, meaning coh, plv, pli, wpli yielded absolute values, while imcoh did not.

### Intraclass Correlation Coefficient

ICCs were calculated with SPSS (version 26 for Macintosh). We used a two-way mixed effects model with absolute agreement, single measurement criteria to estimate ICCs and their 95% confidence intervals. This is often referred to as an ICC (3,1) model. The equation for calculating the ICC is: ICC (3,1) = MS_B_ – MS_E_/MS_B_ + (k – 1) MS_E_, where MS_E_ = Error Mean Square, MS_B_ = Between-subjects Mean Square, and k = number of measurements (34–36). In a two-way mixed effects model, variance consists of 3 components: between-subjects variance (between-subjects mean square), between-tests variance, and random error variance (residual mean squares). Furthermore, by specifying absolute agreement the model is described as: between-subjects variance/(between-subjects variance + between-tests variance + random error variance) (37). ICCs ranged from 0 to 1 with higher values indicating better reliability, any negative values were rescored to zero. Following the guidelines of (7), we defined ICCs as: excellent reliability >0.75, good reliability 0.75–0.60, fair reliability 0.59–0.40, and poor reliability <0.40, similar to (5). ICCs were calculated over 4 timepoints to estimate an average across all 4 runs, and over 2 timepoints to estimate 1 hour and 1 week reliability. In the current study, each rest run was modeled with a fixed effects model, given that identical scanning parameters were used and task familiarity may have occurred. Meanwhile, subjects were modeled with a random effects model, given that sampling and recruitment was random and there was no reason to expect similarity in a spontaneous resting-state task.

### Statistical Analysis

To look at group differences in functional connectivity metrics, data from a single visit (Visit1-Run1, Rest10, a 10 min resting-state task) was analyzed. Analysis of Variance (ANOVA) was performed using SPSS (version 26 for Macintosh) with the between-subjects factor of Group (HC, SZ). Each resting state (eyes open, eyes closed) was analyzed separately. The statistical threshold was set at p < 0.05 for each individual connectivity metric.

## Results

### Whole Brain Reliability

**Figure 2:**
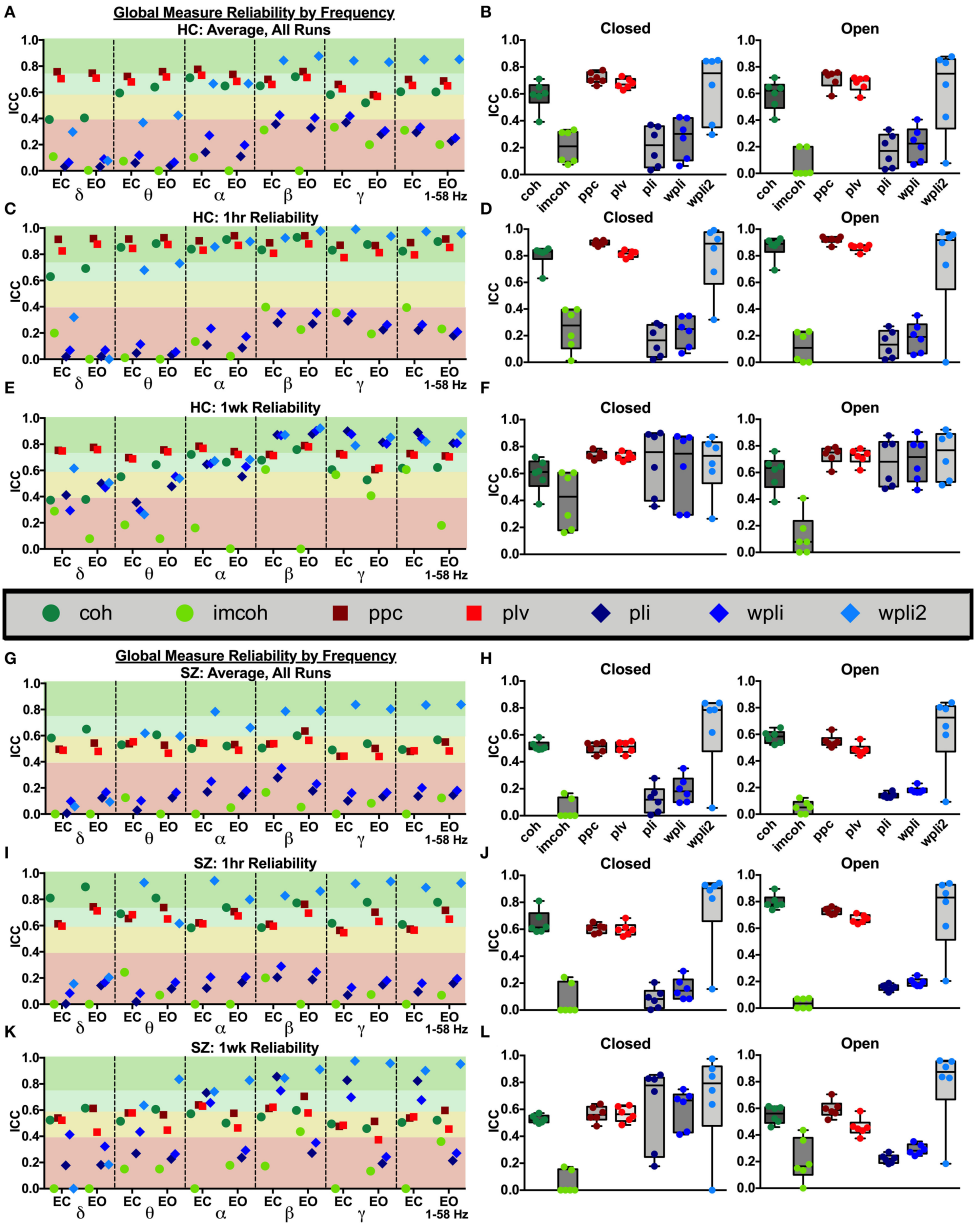
Global reliability. Global ICC estimates for each connectivity measure were calculated in delta-gamma and broadband frequency bands as an average across all runs in HC **(A,B)** and SZ **(G,H)**, across 1 hour in HC **(C,D)** and SZ **(I,J)**, and across 1 week in HC **(E,F)** and SZ **(K,L)**. Data represent mean ICC value. Boxplots contain aggregate information for each connectivity measure in eyes closed and eyes open states.

Connectivity values within all 62 regional labels were averaged to create a whole brain, global reliability measure. As Figure 2 shows, global MEG connectivity reliability varied greatly depending on frequency band, connectivity measure, and participant group examined. Within the broadband (1–58 Hz) average ICC for HC, Figure 2A: there was excellent reliability for wpli2 (ICC_wpli2_ = 0.85), good reliability for ppc, plv, and coh (ICC_ppc_ = 0.69, ICC_plv_ = 0.65, ICC_coh_ = 0.60), and poor reliability for imcoh, pli, and wpli (ICC_imcoh_ = 0.26, ICC_pli_ = 0.26, ICC_wpli_ = 0.29). Within the broadband average ICC for SZ, Figure 2G: there was excellent reliability for wpli2 (ICC_wpli2_ = 0.84), fair reliability for coh, ppc, and plv (ICC_coh_ = 0.53, ICC_ppc_ = 0.52, ICC_plv_ = 0.48) and poor reliability for imcoh, pli, and wpli2 (ICC_imcoh_ = 0.06, ICC_pli_ = 0.14, ICC_wpli_ = 0.19). In both groups, the coh, ppc, and plv had higher reliability across 1 hour than across 1 week, Figures 2C,E for HC and Figures 2I,K for SZ. Also, delta and theta bands generally had lower reliability than alpha, beta, and gamma bands. In HC and SZ, eyes open and eyes closed resting-states had similar reliability levels for most metrics, however, there were a couple metrics in SZ where eyes closed data had higher reliability than eyes open data, for example, 1 week reliability for pli and wpli (EC ICC_pli_ = 0.82 compared to EO ICC_pli_ = 0.21, and EC ICC_wpli_ = 0.68 compared to EO ICC_wpli_ = 0.27), Figures 2K,L.

**Figure 3:**
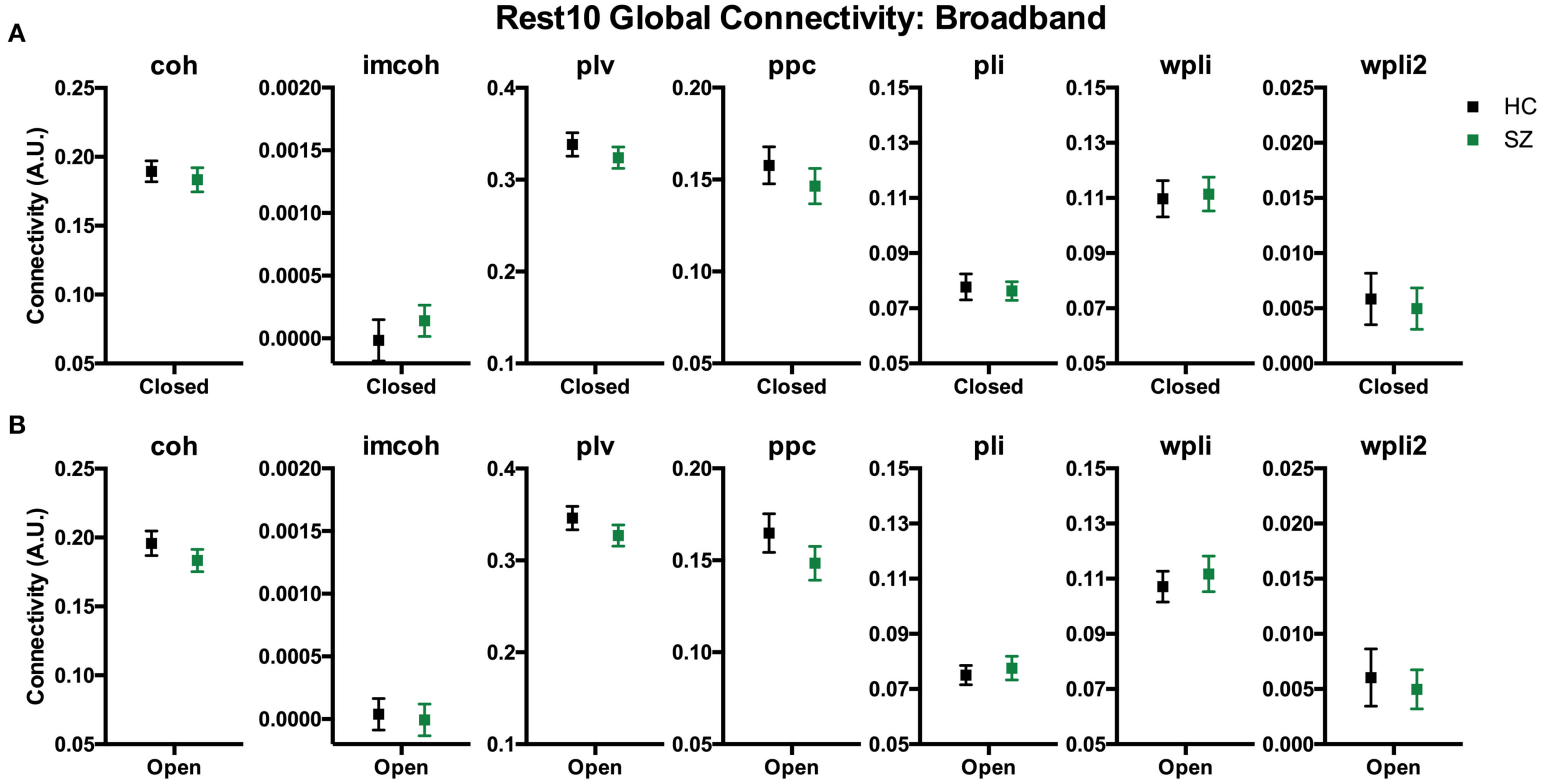
Rest10 global broadband connectivity. Data represent mean (±SEM). Patients with schizophrenia and healthy controls were not significantly different in global broadband connectivity during the eyes closed resting-state **(A)** or eyes open resting-state **(B)**.

To compare whole brain functional connectivity metrics between groups (SZ, HC) the main effect of group was examined during Visit 1 for the Rest 10 task. As shown in Figure 3, there were no significant group effects for any of the global connectivity metrics for broadband (1–58 Hz) frequency, *p*'s > 0.251, suggesting there were no differences between patients with SZ and HC in the 7 functional connectivity metrics at the global level. Individual frequency bands (delta-gamma) were not explored further since broadband analyses did not reveal a group effect in global connectivity.

### Network Reliability

**Figure 4:**
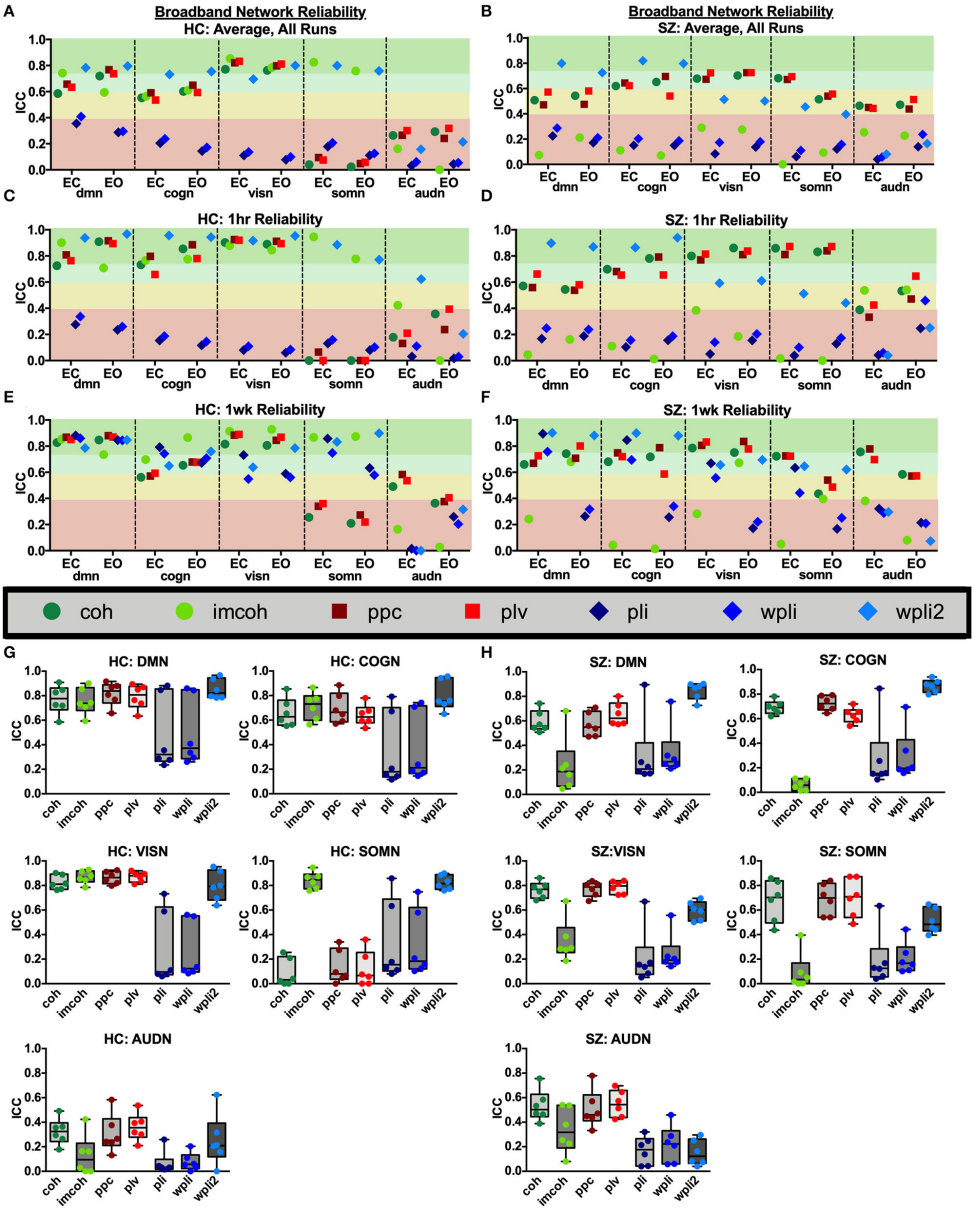
Broadband network reliability. Broadband network ICC estimates for each connectivity measure were calculated for network averages across all runs, for 1 hour and 1 week in the broadband frequency band in HC **(A,C,E)** and SZ **(B,D,F)**. Data represent mean ICC value. Boxplots **(G,H)** contain aggregate information for each connectivity measure within networks.

Broadband connectivity averages within 5 networks, the default mode network (DMN), cognitive control network (COGN), visual network (VISN), somatosensory network (SOMN), and auditory network (AUDN), were examined, as shown in Figure 4. MEG connectivity network reliability varied greatly depending on network, connectivity measure and participant group. For both HC and SZ, the default mode, cognitive control, and visual networks had higher reliability compared to somatosensory and auditory networks. Within the default mode network, Figures 4G,H (DMN), HC had excellent reliability, for coh, imcoh, ppc, plv, and wpli2 (ICC_coh_ = 0.77, ICC_imcoh_ = 0.76, ICC_ppc_ = 0.82, ICC_plv_ = 0.79, ICC_wpli2_ = 0.85) and fair reliability for pli and wpli (ICC_pli_ = 0.48, ICC_wpli_ = 0.50), while SZ had excellent reliability in wpli2 (ICC_wpli2_ = 0.85), good reliability in plv (ICC_plv_ = 0.65), and fair reliability in coh and ppc (ICC_coh_ = 0.59, ICC_ppc_ = 0.57). Within the cognitive control network, Figures 4G,H (COGN), HC had excellent reliability for wpli2 (ICC_wpli2_ = 0.80), and good reliability for coh, imcoh, ppc, and plv (ICC_coh_ = 0.66, ICC_imcoh_ = 0.71, ICC_ppc_ = 0.70, ICC_plv_ = 0.64), while SZ had excellent reliability in wpli2 (ICC_wpli2_ = 0.87), and good reliability in coh, ppc, and plv (ICC_coh_ = 0.69, ICC_ppc_ = 0.72, ICC_plv_ = 0.63). Within the visual network, Figures 4G,H (VISN), HC had excellent reliability for coh, imcoh, ppc, plv and wpli2 (ICC_coh_ = 0.82, ICC_imcoh_ = 0.87, ICC_ppc_ = 0.86, ICC_plv_ = 0.87, ICC_wpli2_ = 0.80), while SZ had excellent reliability for coh, ppc, and plv (ICC_coh_ = 0.76, ICC_ppc_ = 0.77, ICC_plv_ = 0.79) and fair reliability for wpli2 (ICC_wpli2_ = 0.59). Within the somatosensory network, Figures 4G,H (SOMN), HC had excellent reliability for imcoh and wpli2 (ICC_imcoh_ = 0.84, ICC_wpli2_ = 0.83), while SZ had good reliability for coh, ppc, and plv (ICC_coh_ = 0.67, ICC_ppc_ = 0.69, ICC_plv_ = 0.70), and fair reliability for wpli2 (ICC_wpli2_ = 0.51). Within the auditory network, Figures 4G,H (AUDN), SZ had fair reliability for coh, ppc, and plv (ICC_coh_ = 0.53, ICC_ppc_ = 0.51, ICC_plv_ = 0.55). Any measure not listed in the above networks had poor reliability.

**Figure 5:**
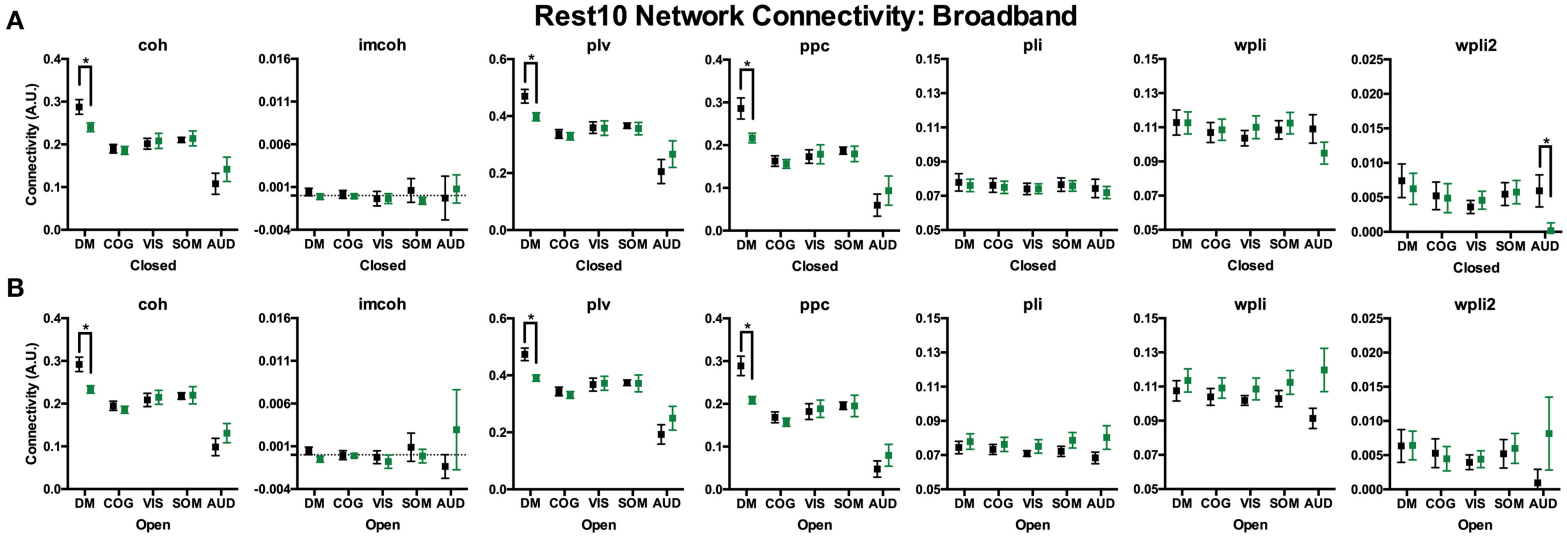
Rest10 network broadband connectivity. Data represent mean (±SEM), asterisks denote p < 0.05. Patients with schizophrenia had reduced broadband default mode network average coherence, phase-locking value, and pairwise phase consistency during the eyes closed resting-state **(A)** and eyes open resting-state **(B)**. Patients with schizophrenia also had reduced broadband auditory network average weight phase lag index debiased during the eyes closed resting-state **(A)**.

To compare broadband network functional connectivity metrics between groups (SZ, HC) the main effect of group was examined during Visit 1 for the Rest 10 task. As shown in Figure 5, SZ had significantly reduced coh, plv, ppc in the dmn, when compared to HC, during both eyes closed [Group effect for coh: *F*(1,24) = 5.631, *p* = 0.026, plv: *F*(1,24) = 6.766, *p* = 0.016, ppc: *F*(1,24) = 6.564, *p* = 0.017] and eyes open [Group effect for coh: *F*(1,24) = 9.464, *p* = 0.005, plv: *F*(1,24) = 11.172, *p* = 0.003, ppc: *F*(1,24) = 10.864, *p* = 0.003] resting states, Figures 5A,B, respectively. Furthermore, SZ had significantly reduced wpli2 in the audn during an eyes closed resting state [Group effect for wpli2: *F*(1,24) = 5.001, *p* = 0.035].

**Figure 6:**
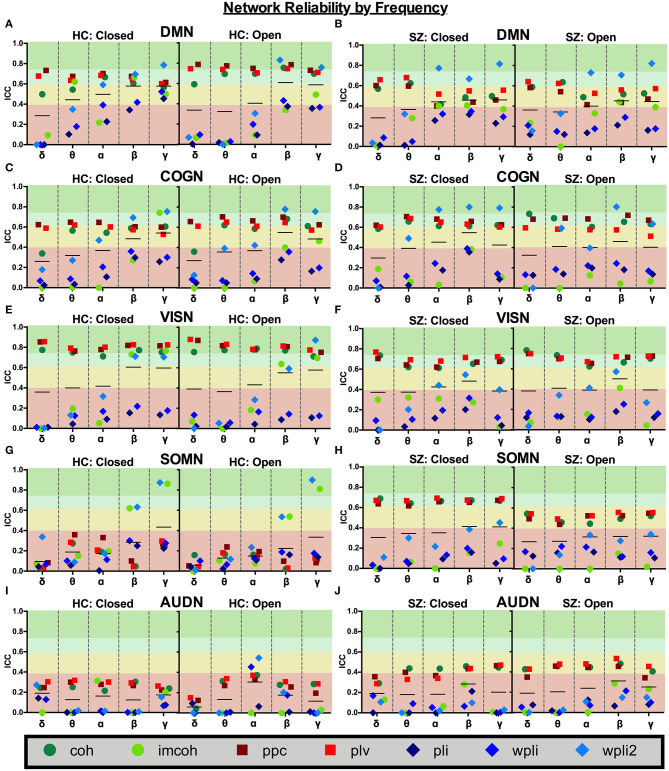
Network reliability across frequency. Network ICC estimates for each connectivity measure were calculated as an average across all runs for each frequency band (delta-gamma) and in each resting state (eyes closed and eyes open). Network averages for DMN **(A,B)**, COGN **(C,D)**, VISN **(E,F)**, SOMN **(G,H)**, and AUDN **(I,J)** are shown in HC and SZ, respectively. Data represent mean ICC value.

Average network reliability within frequency bands is shown in Figure 6. To help determine which measurement time (1 hour vs. 1 week) drove the average, Supplementary Figures 1, 2 further break down network reliability within frequency bands for 1 hour and 1 week. An alternative version of Figure 6, showing variability across networks is also provided in Supplementary Figure 3. As with broadband data, the somatosensory and auditory networks across all frequencies had the lowest reliability. Similar reliability levels were found in both resting-states for all metrics (average HC mean difference = 0.01, SZ mean difference = 0.02).

### Regional Pair Reliability

**Figure 7:**
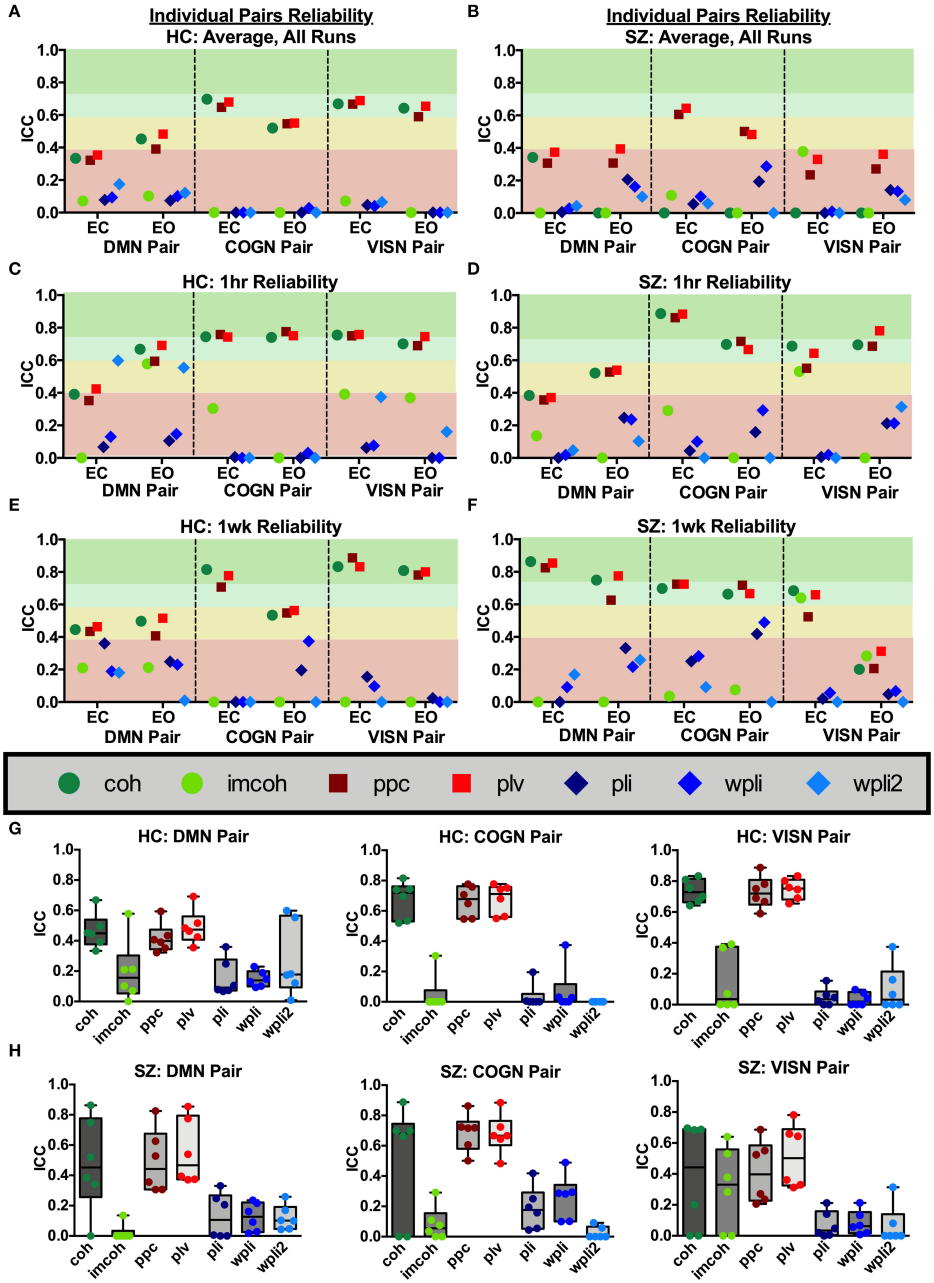
Network pair reliability. Network pair ICC estimates for each connectivity measure were calculated as an average across all runs, for 1 hour and 1 week in the broadband frequency band in HC **(A,C,E)** and SZ **(B,D,F)**. Data represent mean ICC value. Boxplots **(G,H)** contain aggregate information for each connectivity measure within each network pair.

Broadband connectivity metrics within 3 individual network pairs (DMN pair, COGN pair, VISN pair) were examined, as shown in Figure 7. MEG connectivity network reliability varied greatly depending on network pair, connectivity measure and participant group. Within the default mode network pair, Figures 7G,H (DMN Pair), HC had fair reliability for coh, ppc, and plv (ICC_coh_ = 0.46, ICC_ppc_ = 0.42, ICC_plv_ = 0.49), and SZ had fair reliability for coh, ppc, and plv (ICC_coh_ = 0.48, ICC_ppc_ = 0.49, ICC_plv_ = 0.55). Within the cognitive control network pair, Figures 7G,H (COGN Pair), HC had good reliability for coh, ppc, and plv (ICC_coh_ = 0.68, ICC_ppc_ = 0.66, ICC_plv_ = 0.68), while SZ had good to fair reliability for coh, ppc, and plv (ICC_coh_ = 0.49, ICC_ppc_ = 0.69, ICC_plv_ = 0.68). Within the visual network pair, Figures 7G,H (VISN Pair), HC had good reliability for coh, ppc, and plv (ICC_coh_ = 0.73, ICC_ppc_ = 0.73, ICC_plv_ = 0.75), while SZ had fair reliability for ppc, and plv (ICC_ppc_ = 0.41, ICC_plv_ = 0.51). Any measure not listed in the above networks had poor reliability. For both groups, the connectivity metrics imcoh, pli, wpli, and wpli2 had poor reliability in all 3 network pairs tested. Eyes open and eyes closed had similar reliability levels in HC and SZ for all metrics (average HC mean difference = 0.01, SZ mean difference = 0.01).

**Figure 8:**
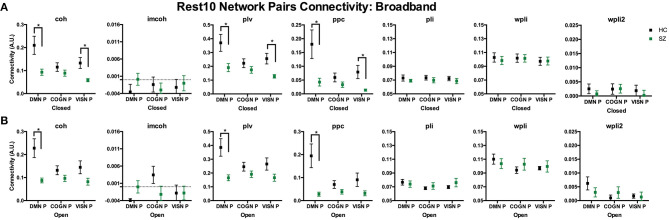
Rest10 network pairs broadband connectivity. Data represent mean (±SEM), asterisks denote *p* < 0.05. Patients with schizophrenia had reduced default mode network pair (right precuneus to left medial orbitofrontal cortex) and visual network pair (left lateral occipital to right middle temporal) coherence, phase-locking value, and pairwise phase consistency during the eyes closed resting-state **(A)**. Patients with schizophrenia had reduced default mode network pair (right precuneus to left medial orbitofrontal cortex) coherence, phase-locking value, and pairwise phase consistency during the eyes open resting-state **(B)**.

To compare regional pair functional connectivity metrics between groups (SZ, HC) the main effect of group was examined during Visit 1 for the Rest 10 task. As shown in Figure 8, SZ had significantly reduced coh, plv, ppc in the dmn connectivity pair (precuneus right hemisphere to medial orbitofrontal cortex left hemisphere), during both eyes closed [Group effect for coh: *F*(1,24) = 7.817, *p* = 0.010, plv: *F*(1,24) = 6.891, *p* = 0.015, ppc: *F*(1,24) = 6.518, *p* = 0.017] and eyes open [Group effect for coh: *F*(1,24) = 11.071, *p* = 0.003, plv: *F*(1,24) = 10.789, *p* = 0.003, ppc: *F*(1,24) = 10.154, *p* = 0.004] resting states when compared to HC, Figures 8A,B, respectively. Furthermore, SZ had significantly reduced coh, plv, ppc in the visn connectivity pair (lateral occipital left hemisphere to middle temporal right hemisphere) during an eyes closed resting state, [Group effect for coh: *F*(1,24) = 9.004, *p* = 0.010, plv: *F*(1,24) = 10.132, *p* = 0.004, ppc: *F*(1,24) = 7.345, *p* = 0.012] Figure 8A, when compared to HC.

## Discussion

Following source analysis various FNC metrics were compared, specifically coh, imcoh, ppc, plv, pli, wpli, and wpli2. The reliability of these metrics varied greatly depending on frequency band, network, network pair, and participant group examined. To summarize a few key findings: (1) Broadband whole brain averages in both HC and SZ showed excellent reliability for wpli2, good to fair reliability for ppc, plv, and coh and poor reliability for imcoh, pli, and wpli, (2) Network averages showed excellent to good reliability for coh, imcoh, ppc, plv, and wpli within default mode, cognitive control, and visual networks in HC, while the same metrics had excellent to fair reliability in SZ, (3) Regional network pair averages showed good to fair reliability for coh, ppc, and plv within default mode, cognitive control and visual network pairs, while imcoh, pli, wpli, and wpli2 all had poor reliability, (4) For both HC and SZ, the default mode, cognitive control, and visual networks had higher reliability compared to somatosensory and auditory networks, and (5) Eyes open and eyes closed states had similar reliability levels in HC and SZ for all metrics. When taken together, the results indicate functional connectivity reliability is highly dependent on connectivity metric, frequency band, and region or network size.

In HC, we confirmed some patterns of functional connectivity for certain metrics and frequency bands, which were in line with previous research. Our results in HC were fairly consistent with previous resting-state MEG studies, although there were a few differences. For example, in MEG whole-brain functional connectivity comparisons plv has been found to range from excellent to good reliability (ICC range 0.74–0.82) in alpha-gamma bands, while pli has been found to have poor reliability (ICCs < 0.1) for all frequency bands in both eyes open and eyes closed resting-states (2). Here, we found similar results that global functional connectivity averages for plv had excellent to good reliability across 1 hour and 1 week in delta-gamma bands and in broadband (ICC_plv_ = 0.65), while pli had poor reliability in all bands across 1 hour and in broadband (ICC_pli_ = 0.26). We also found that both eyes open and eyes closed resting states had similar reliability levels. However, in contrast to the previous paper, we found that pli had excellent reliability in beta, gamma, and broadband across 1 week. Another previous study examining MEG global whole brain reliability found that coh and wpli had good to excellent reliability in delta-gamma bands (4). In contrast to this, the present study found that imcoh and wpli generally had poor reliability in all frequencies with a few exceptions, such as fair to good reliability for imcoh during an eyes closed state in beta, gamma, and broadband and fair to excellent reliability for wpli across 1 week. A potential difference between the two studies may be how imcoh was calculated. Here, we reported the imcoh measure without using absolute values, which is the default formula used by MNE software and the original publication (28). To get undirected connectivity for imcoh, certain source models benefit from using imcoh absolute values, as (4) did. At the resting-state network level, there is often poor reliability in phase or coh based metrics which are robust to spatial leakage artifacts, such as pli and its derivatives, as well as imcoh (1). We found similar results here and reported that our network averages showed lower reliability in metrics robust to spatial leakage artifacts, e.g., imcoh, pli, wpli, and wpli2. It is interesting that both results showed low reliability for the metrics considering that we used an anatomical parcellation and dSPM algorithm, whereas the previous publication used a data-driven parcellation from fMRI along with a beamformer algorithm. Poor reliability of phase based metrics has also been seen in another MEG study which found poor reliability in pli in all frequency bands and networks, but good to excellent reliability in plv in alpha-gamma bands for visual, sensorimotor, auditory, and default mode networks (2). Here, we found excellent to good reliability for coh, plv, and ppc within default mode, cognitive control, and visual networks, with mixed reliability for pli and wpli dependent on network and interval length (1 hour vs. 1 week). Although graph analysis was not used in the current study, it should be mentioned that the reliability of those derived resting-state MEG functional connectivity networks have also been variable, ranging from poor to good (ICCs 0.256–0.655) depending on band and metric defining nodal centrality, with greatest reliability in eyes open resting state networks when assessed with Dnodal and Enodal metrics (38). Further direct comparison between our research and (1, 2, 4) is difficult to interpret given that each study used different source analysis and network modeling methods. Functional connectivity metrics based on phase-related connectivity can minimize the impact of spatial leakage and zero-lag synchronization, however, the estimates may be more variable in short or noisy recordings, or across connectivity pair data. It has been previously suggested that amplitude envelope correlation and partial correlation measures have higher reliability and are the most consistent functional connectivity methods for an MEG resting-state (1), however, those metrics were not tested in the current study. Phase-related connectivity metrics perform better when averaged across larger brain regions, more voxels, and in larger datasets, as sample size negatively impacts pli and imcoh metrics.

Magnetic field spread or spatial leakage artifacts is a problem in MEG functional connectivity estimation (39–41) that can influence measures of functional connectivity and artificially inflate reliability (1); therefore, metrics which avoid those confounds, such as imcoh, pli, wpli, phase slope index, amplitude envelope correlation, are generally recommended. However, the confounds introduced into connectivity estimation due to spatial leakage have been shown to be highly repeatable across scans and between subjects (1). Here, we also found that the metrics which are prone to spatial leakage, e.g., coh, plv, and ppc, generally have higher repeatability or reliability across sessions. The higher reliability may be spurious in nature, but coh, plv, and ppc remained consistently higher in reliability even when region size and number of regions averaged fluctuated in our data, e.g., throughout global, network, and regional pair data.

While signal leakage is expected to be highly reliable and may on the surface appear to influence the reliability of certain connectivity metrics, there are two additional factors which indicate that the reliability of coh, ppc, and plv is not solely attributed to signal leakage. First, signal leakage, especially for MEG, does not spread across the entire brain but remains relatively localized (41). In our data, the regional network pairs we selected represented “distant” intra network sources, e.g., parietal to anterior frontal or left occipital to right temporal, greatly decreasing the likelihood of the increased reliability of the coh, ppc and plv metrics to be influenced by signal leakage alone. Importantly also, these metrics that cannot directly eliminate the possibility of signal leakage also retain additional signal (zero-phase correlations) that metrics robust to signal leakage ignore. Invasive measures have demonstrated zero-phase correlations across broad regions of the brain indicating that not all zero-phase correlations are related to artifact alone, but contain real signal; therefore, eliminating all zero-phase correlations may reduce reliability by removing signal. There are other spatial leakage correction methods for MEG, besides removing zero-phase correlations, which may improve reliability, such as geometric correction scheme which removes spurious local connections without impacting dynamic hub regions and networks at rest (42) or adaptive cortical parcellations (43). In fact, it has been suggested that using a non-zero-lag connectivity metric does not obviate the need for adaptive parcellation. Based on this information, we consider the current results to support the reliability of coh, ppc, and pli across region, network and whole brain analyses.

Interestingly, our research found that the non-zero-lag connectivity metrics pli, wpli, and wpli2 had variable reliability depending on the size and number of regions averaged (i.e., as spatial resolution was increased, reliability decreased). Those metrics had their highest reliability in global averages, followed by network averages, and lowest reliability in individual network pair connections. For example, when wpli2 was not averaged across the whole brain or across a network, but in an individual network pair, HC and SZ groups both had poor reliability (HC ICC Avg = 0.14, SZ ICC Avg = 0.11), similar to previous results (5), yet global averages with all regions showed excellent reliability (HC ICC Avg = 0.85, SZ ICC Avg = 0.84). Even within network averages, the networks which contained more regions (default mode-14 regions, cognitive control-26 regions, and visual-12 regions) had higher reliability than networks defined by fewer regions (somatosensory-8 regions and auditory-2 regions). While some have used 2 or 3 nodes to characterize a resting-state network (2), we decided to use networks defined by the fMRI ICA resting-state network approach (12, 29, 44), a technique which has been successfully applied to MEG resting-state networks in SZ clinical populations (11, 14, 15).

Here, we showed several instances where patients with SZ had lower reliability in functional connectivity metrics, e.g., lower broadband whole brain averages and network averages when compared to HC. In our previous test–retest paper, which only examined wpli2 in select superior parietal regional connectivity pairs, we also found poor reliability in the metric for patients with SZ (ICC = 0.03), as well as HC (ICC = 0.12) (5). Although reliability was low, meaning significant effects were not consistent across run, we found instances of increased functional connectivity between superior parietal to lateral occipital and superior parietal to entorhinal connections in patients with SZ (5). However, these results may not replicate because the metric is unreliable. Previous research has shown that abnormal resting-state functional connectivity is a key process underlying SZ (9). While there are no other test–retest functional connectivity reliability studies to directly compare to, one study used imcoh and found decreased alpha-band connectivity in left prefrontal cortex and right superior temporal cortex together with increased connectivity in left extrastriate cortex and right inferior prefrontal cortex in patients with SZ (10). Our research would suggest that imcoh is a connectivity metric with low reliability in this patient population. We also found higher variability in ICC 95% confidence intervals (data not shown) in patients with SZ suggesting greater between subjects variability, similar to the dynamic functional connectivity finding that patients change meta-states more often than HC and exhibit greater inter-individual variability (14). Despite the difficulties in interpreting the functional meaning of lower reliability and higher variability in patients with SZ, the current findings are consistent with deficits in functional connectivity and neural oscillations previously reported (5, 11, 14, 16, 39, 45). Aside from differences in ICC, here, we also found that patients with SZ had significantly reduced coh, plv, and ppc metrics in the default mode network average and pair (right precuneus to left medial orbitofrontal cortex) values when compared to HC, in the Rest10 task during Visit 1. We also found reductions for patients with SZ in coh, plv, and ppc metrics in the visual network pair, and for wpli2 in the auditory network during an eyes closed resting-state. When combined with the information that ICCs for these metrics (coh, plv, ppc) were relatively high, it implies the reduction in default mode functional connectivity seen in patients with SZ is somewhat stable during both eyes open and eyes closed resting-states.

A key question in examining test–retest reliability of resting state networks with MEG is whether there are stable networks within the time window assessed. Simulations have shown that at the network level, only longer window lengths were sufficient to detect resting-state networks that matched the ground truth, especially for plv, amplitude envelope correlation, and coh (46). While fMRI has presented multiple studies demonstrating the reliability of connectivity between different regions, the timescale of MEG is different and may present as more or less reliable depending on how these connectivity patterns are assessed. However, the visual occipital alpha activation that shows often reliable patterns of activation when changing between eyes open and eyes closed within subject provide evidence for the reliability of oscillatory networks. An additional question that remains is why clinical populations may exhibit different test–retest reliability than HC. A core characteristic of SZ is that patients experience repeated relapses even after initiation of medication (47). This supports the general idea that brain dynamics in patients are more variable and is consistent with the general hypothesis that healthy brain dynamics are maintained through homeostasis and deviations from this stable state lead to functional consequences (48). Future research is needed to determine if this reduction in reliability of measures in patients with SZ is dependent on medication, disease severity or disease duration and may further inform clinical treatment.

The current study was designed to compare available functional connectivity metrics in a test–retest dataset of patients with SZ and HC. We reported connectivity values without modification from the MNE provided functions and used a surface-based source space with a fixed surface orientation. However, it should be noted that certain functional connectivity metrics, e.g., imcoh, can become difficult to interpret when source direction is not well-defined. The other metrics used (coh, plv, ppc, pli, wpli, wpli2) are the result of absolute value calculations which account for sign flips across sessions. Others using imcoh should carefully evaluate their models to avoid introducing extra variability.

There are several limitations in the present study which warrant caution. The patient population recruited was a stable, medicated cohort of patients with SZ. As such, the results may not generalize to a more varied group of individuals with psychosis, other populations and/or imaging sites. Furthermore, it remains unknown if the functional connectivity abnormalities found were due to underlying neurophysiology of schizophrenia or were driven by medication, as all patients were antipsychotic medications. Another cautionary note is the small sample size. Although ICCs were calculated across 4 separate runs, the small group size (*n* = 13) warrants caution when generalizing to larger samples. Also, it is important to consider the ICC model itself. An ICC examines variance changes within and between subjects over time. Occasionally, a low ICC can reflect that a within-subject change occurred, and may not imply that a measure itself is inaccurate. While results between our study and others are similar, each study modeled ICC estimates differently and ICC values will fluctuate based on the model and variance assumptions (35, 36, 49). Another aspect to consider is the localization algorithm used. The optimal source localization algorithm to examine functional connectivity remains to be determined. One advantage of the dSPM algorithm is that its assumptions do not limit the ability to capture synchronous activity, which remains a limitation of most implementations of the beamformer approach. However, the dSPM algorithm is also known to have limited spatial resolution and also can propagate noise throughout the brain. As such, using dSPM may impact the sensitivity of the functional connectivity metrics measured. Future studies should examine realistic simulated connectivity patterns to determine the conditions under which the best results are obtained. Finally, the current study included a single MEG system, definitive conclusions on reliability cannot be made until a larger sample size and multiple sites are included.

Our research demonstrates that resting-state connectivity in clinical populations can be informative and reliable. Certain functional connectivity metrics should be preferred due to their higher reliability. MEG can be used to capture neural oscillatory networks in resting-states with good spatial precision and reliability. Both eyes open and eyes closed resting states were reliable over sessions and should be reported to best capture neural dynamics.

## Data Availability Statement

The original contributions generated for the study are included in the article/Supplementary Files, further inquiries can be directed to the corresponding author.

## Ethics Statement

The studies involving human participants were reviewed and approved by University of New Mexico Health Sciences Center Human Research Review Committee. The patients/participants provided their written informed consent to participate in this study.

## Author Contributions

FC-C and JS: design and writing. FC-C: data collection and processing, formal analysis, and funding acquisition. JS: supervision. Both authors contributed to and have approved the final manuscript.

## Conflict of Interest

The authors declare that the research was conducted in the absence of any commercial or financial relationships that could be construed as a potential conflict of interest.
