# Supplementary material for: Test–Retest Reliability of Magnetoencephalography Resting-State Functional Connectivity in Schizophrenia
Source: Front Psychiatry. 2020 Dec 16;11:551952. doi: 10.3389/fpsyt.2020.551952 (PMC7772354; doi:10.3389/fpsyt.2020.551952)
Supplement: Supplementary file 2 [file Data_Sheet_2.docx]

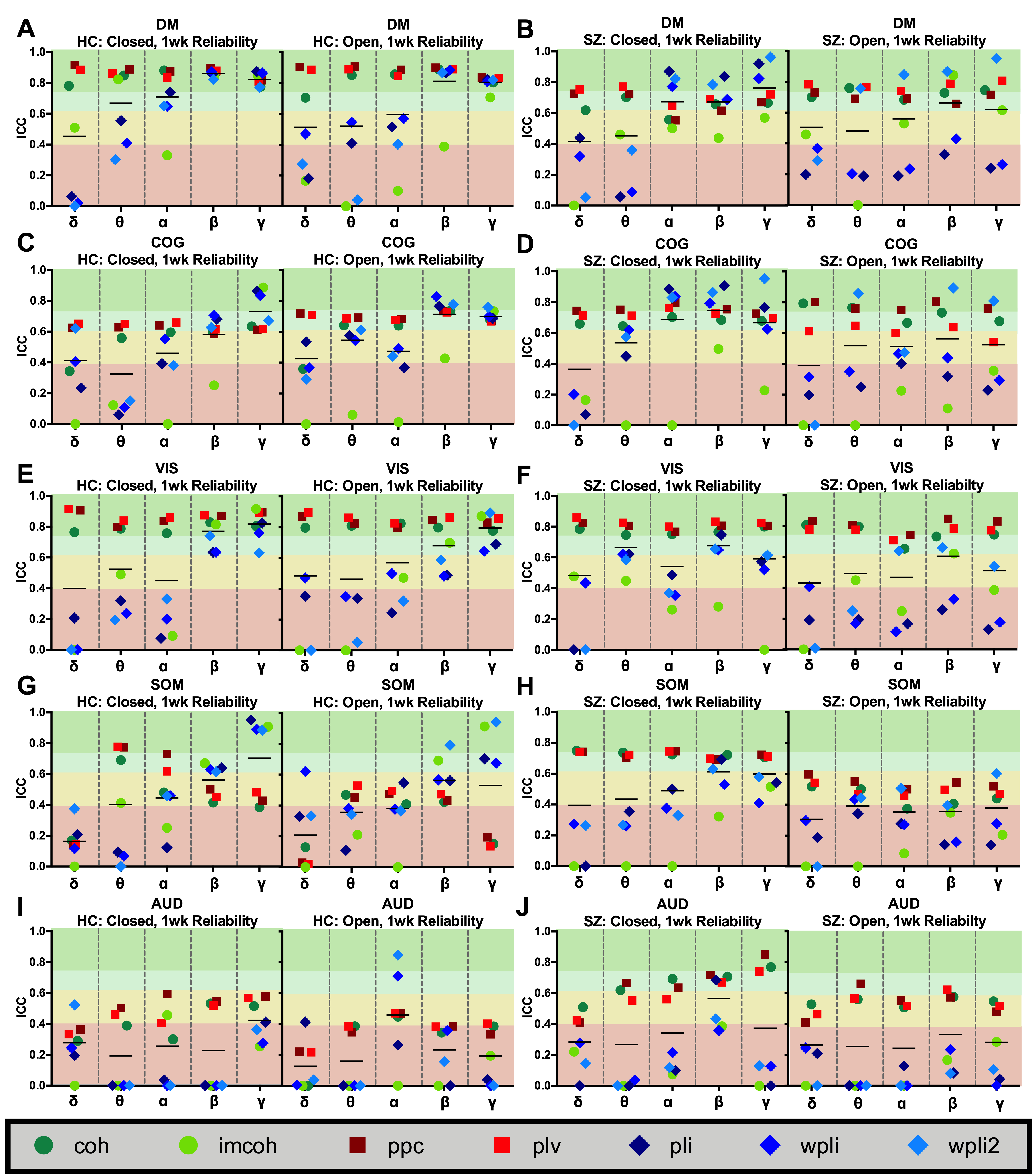


**Supplementary Figure 2.** **One week network reliability across frequency bands.** Network ICC estimates for each connectivity measure were calculated for one week reliability for each frequency band (delta-gamma) and in each resting state (eyes closed and eyes open). Data represent mean ICC value.
